# The effect of a six-week osteopathic visceral manipulation in patients with non-specific chronic low back pain and functional constipation: study protocol for a randomized controlled trial

**DOI:** 10.1186/s13063-018-2532-8

**Published:** 2018-03-02

**Authors:** Walkyria Vilas Boas Fernandes, Cleofás Rodríguez Blanco, Fabiano Politti, Fernanda de Cordoba Lanza, Paulo Roberto Garcia Lucareli, João Carlos Ferrari Corrêa

**Affiliations:** 1 Doctoral Program in Rehabilition Sciences, Nove de Julho University (UNINOVE), São Paulo, Brazil; 2 Federal University of Mato Grosso (UFMT), Rondonópolis, Brazil; 3 Doctoral Program in Health Sciences, Seville University, Sevilla, Spain; 4 Human Movement Analysis Laboratory, Nove de Julho University (UNINOVE), São Paulo, SP Brazil

**Keywords:** Visceral manipulation, Low back pain, Constipation, Flexion-relaxation, Electromyography

## Abstract

**Background:**

The aim of the proposed study is to analyze the effect of a six-week osteopathic visceral manipulation (OVM) program on the flexion-relaxation phenomenon in individuals with non-specific chronic low back pain (LBP) and functional constipation.

**Methods/Design:**

An assessor-blinded, two-arm, randomized, placebo-controlled trial will be conducted. The sample will comprise 76 individuals with non-specific chronic LBP who have functional intestinal constipation, aged 18–65 years. The participants will be randomly allocated to two groups: (1) OVM and (2) sham OVM (SOVM). Evaluations will involve an interview, the Oswestry Disability Index, Fear-Avoidance Beliefs Questionnaire, functional constipation according to Rome III criteria, Biering-Sorensen test to normalize electromyographic (EMG) data, T12–L1 paraspinal level of the EMG signal during the flexion-relaxation phenomenon, 11-point numeric pain rating scale and fingertip-to-floor test. OVM and SOVM will be performed once per week for six weeks. Group 1 will receive OVM for 15 min and Group 2 will receive a sham visceral technique. Evaluations will be performed before and after the first session, after six weeks of treatment, and three months after randomization (follow-up). The findings will be analyzed statistically considering a 5% significance level (*p* ≤ 0.05). The limitation of the study is that the therapist will not be blinded.

**Discussion:**

This will be the first trial to analyze the clinical response and electromyographic signals during the flexion-relaxation phenomenon after OVM.

**Trial registration:**

Brazilian Clinical Trial Registry, RBR-7sx8j3. Registered on 26 October 2017.

**Electronic supplementary material:**

The online version of this article (10.1186/s13063-018-2532-8) contains supplementary material, which is available to authorized users.

## Background

Recent research shows that low back pain (LBP) can cause more years of disability than any other health condition [1]. Chronic pain is a public health problem, as it is an important cause of morbidity, work absenteeism, and temporary or persistent incapacity, generating high costs for healthcare systems [2]. There is an increasing demand for the treatment of chronic LBP [3] and researchers report that 80–90% [4–6] of cases are classified as non-specific LBP.

LBP is considered the second most common reason for visits to first-contact practitioners, such as chiropractors and osteopaths [7]. Besides using spinal manipulation [8–10], these professionals also employ visceral techniques [11] with a conservative approach. The theory is that visceral disorders could potentially trigger or exacerbate LBP symptoms due to impaired movement between internal organs and respective supporting tissues. This could manifest as LBP through two possible mechanisms: referred visceral pain and central sensitization [11].

Studies have shown that visceral techniques applied to healthy individuals lead to an immediate increase in the pain threshold of the low back compared to placebo application [12]. Researchers have also studied specific visceral disorders, such as refractory irritable bowel syndrome [13] and chronic constipation in women [14], and found better results after visceral treatment. While some researchers have performed visceral techniques on patients with LBP [11, 15, 16], the physiological and biomechanical mechanisms remain untested.

There is evidence that patients with LBP have deficits in the neuromuscular control of the spine [17–19] and that electrical activity of the trunk muscles can be used to evaluate the effects of therapeutic interventions [19–21] as well as differentiate individuals with LBP, as such individuals have higher electromyographic signals compared to asymptomatic individuals [17, 18, 22–24]. However, it is not known whether the abnormal electromyographic (EMG) activity in the paraspinal muscles of patients with LBP is the cause or consequence of pain [24, 25].

Individuals with chronic LBP do not reach the flexion-relaxation phenomenon (FRP), which is the decrease in or absence of electromyographic activity in the paraspinal muscles found during full trunk flexion in asymptomatic individuals [17–19]. In patients with LBP, the absence of this phenomenon may be due to muscle spasms, decreased range of motion, exaggerated stretch reflexes, or the protection of injured passive structures [26].

Based on the literature, there are indications that the FRP may be a valuable clinical tool to assist in the diagnosis and treatment of patients with LBP [17, 18, 24, 27] and there have been very few studies on the use of visceral techniques for such patients. Thus, the aim of the proposed study is to determine whether osteopathic visceral manipulation (OVM) can modulate stabilizing neuromuscular responses of the lumbar spine and reduce both pain intensity and disability in individuals with non-specific chronic LBP and functional intestinal constipation.

### Primary objective

The primary objective of the proposed study is to analyze the effect of a six-week OVM program on pain intensity and the disability index in individuals with non-specific chronic LBP and functional intestinal constipation.

### Secondary objective

The secondary objective of the proposed study is to analyze the effect of a six-week OVM program on EMG signals of paraspinal muscles during the FRP, the global flexibility, and the fear-avoidance beliefs in individuals with non-specific chronic LBP and functional intestinal constipation.

### Hypothesis

The authors hypothesize that the group submitted to OVM will experience more beneficial effects compared with similar individuals who receive placebo visceral techniques.

### Study design

An assessor-blinder, two-arm, placebo-controlled RCT will be conducted.

## Methods/Design

### Sample selection

Individuals with non-specific chronic LBP will be recruited from physical therapy clinics in the city of Rondonópolis, state of Mato Grosso, Brazil and will be selected based on the eligibility criteria listed below.

### Inclusion criteria

- Age 18–65 years [28]
- Non-specific LBP for at least three months [28]
- Pain intensity of at least 2 points measured using the Numeric Pain Rating Scale [11]
- Functional constipation according to Rome III criteria [29]

### Exclusion criteria

- Any contraindication to OVM or having undergone treatment in the previous six months
- Having undergone spinal surgery in the previous six months
- Serious spinal pathology (e.g. metastasis, spinal fracture, inflammatory, and infective diseases, caudal equine syndrome, canal stenosis)
- Serious cardiovascular or metabolic disease
- Pregnancy
- Red flag signals [5]
- Currently in an acute inflammatory phase of known gastrointestinal or urinary diseases (such as cholecysticis, renal calculi, peritonitis, appendicitis)

### Intervention

The participants will be allocated to groups receiving one of two interventions: (1) OVM or (2) sham OVM (SOVM). The participants in each group will receive six sessions (one per week for six weeks) (Table 1). Given the nature of the study, it is not possible to blind the therapist, but the assessor and patients will be blinded to the treatment conditions. For ethical reasons, the patients in both groups will receive an information booklet called The Back Book in Portuguese [30] on the first day of treatment.

### Group 1: osteopathic visceral manipulation

This group will receive OVM (15 min per session, one session per week for six weeks). The OVM techniques that will be used are described by Ricard [31] and will be performed by a single osteopath with more than ten years of experience. In the first part of each consultation, all patients will be submitted to a direct visceral evaluation [12]. Each treatment will be individualized for each patient using specific visceral manipulation techniques [11, 16] involving light or deep manual fascial releases as well as specific small and large intestine mobilizations in the abdomen, as appropriate [31].

### Group 2: sham technique

This group will receive SOVM at the same time as Group 1 (15 min per session, one session per week for six weeks), which will involve just light touches over the different parts of the abdomen, without any deep mobilization or movement. The osteopath will apply her hands over the same points with the same duration as in OVM to give the patient the perception of being treated [11–13, 15].

### Outcome measures

A blinded assessor will record outcome measures.

The primary outcomes will be LBP intensity (NPRS) and the Oswestry Disability Index (ODI) after the six weeks of treatment and three months after randomization, because pain is the most common reason patients seek private physical therapy clinics for the treatment of LBP. From a patient’s perspective, it is also the outcome that most determines whether treatment has been successful [32].

The secondary outcomes will be the EMG signals during the FRP and fingertip-to-floor test (FFT) after the first treatment session, after the six weeks of treatment and three months after randomization and the FABQ after the six weeks of treatment and three months after randomization.

### Participants’ timeline

**Fig. 1:**
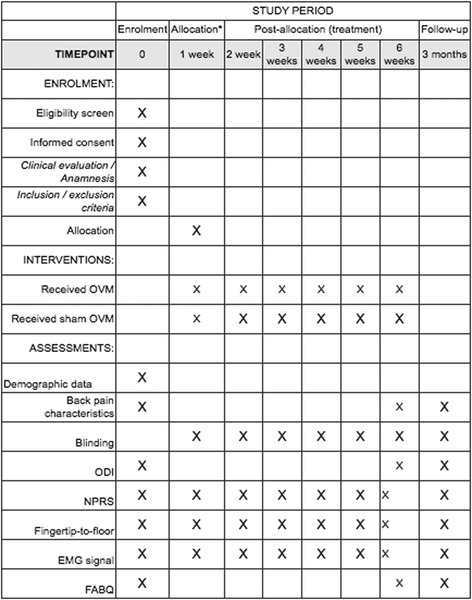
Standard Protocol Items: Recommendations for Interventional Trials (SPIRIT) figure for patient participations. ODI Oswestry Index Disability, NPRS Numeric Pain Rating Scale, EMG electromyographic, FABQ Fear-Avoidance Beliefs Questionnaire

A brief Standard Protocol Items: Recommendations for Interventional Trials (SPIRIT) flow diagram is provided in Fig. 1, and a populated SPIRIT checklist is provided in Additional file 1.

**Table 1:** Items included in the Template for Intervention Description and Replication (TIDieR) checklist: information to include when describing an intervention. Full version of checklist provides space for authors and reviewers to give location of the information

Item no.	Item
Brief name	
1	OVM
Why	
2	Visceral osteopathy is described as a manual treatment performed directly on the viscera with the goal to normalize the mobility dysfunction of the organ and try to eliminate fascial restrictions and relax the visceral spasms.
What	
3	As previously described, the treatment with OVM is manual therapy, because of this it is performed only with the hands.
4	Manual techniques will be performed in the small and large intestine of the volunteers. At the beginning of each session, individuals will be assessed and points of spasms of the visceral musculature of the small and large intestine or fascial restriction will be located. All the techniques are described in the visceral osteopathy book [31].
Who provided	
5	The physiotherapist who will perform the treatment is graduated in physiotherapy for 13 years and she has > 10 years of experience in this type of treatment. She is an osteopath certified by Madrid’s Osteopathy School (Escuela de Osteopatía de Madrid).
How	
6	Six individual weekly sessions of 15 min each will be carried out.
Where	
7	The sessions will be carried out in a private clinic in the city of Rondonópolis/MT.
When and how long	
8	The participants will receive six individual weekly sessions. The duration will be 15 min for each session.
Tailoring	
9	At the beginning of each session, the volunteers will be evaluated according to visceral spasms in the large and small intestine and fascial restriction for manual treatment according to the need of each patient. After the evaluation, the patients will be treated with techniques of visceral osteopathy in the large and small intestine to normalize the visceral musculature spasms.
Modifications	
10^a^	–
How well	
11	Osteopaths are trained to identify visceral spams and/or fascial restriction when they do osteopathy’s formation. We try to propose a treatment respecting the individuality of the patients and seeking to reproduce better the clinical practice. As previously proposed by Panagoupols et al. [11] and Tamer et al. [16]).
12^a^	–

^a^If a checklist is completed for a protocol, these items are not relevant to protocol and cannot be described until the study is complete

### Sample size calculation

The sample size was calculated using G Power 3.1.9.2 software. This calculation was based on the detection of a 10-point difference on the ODI and 2.5-point difference on the NPRS, which have been identified as the minimum clinically important differences [33]. A sample size of 32 participants per group would provide a 90% power to detect a clinically important difference between groups, assuming a common standard deviation of 12 on the ODI [34] and 3.0 on the NPRS and a two-sided hypothesis with an alpha level of 0.5. The sample will be increased by 20% to compensate for possible dropouts, leading to 38 individuals in each group (overall sample = 76 participants).

### Recruitment

The patients will be interviewed by the blinded assessor, who will determine eligibility. Eligible patients will receive clarifications regarding the objectives of the study and will be asked to sign a statement of informed consent. Sociodemographic data and medical history will then be recorded. The assessor will collect the data related to the study outcomes at baseline, before and after a single treatment session, after the completion of the six weeks of treatment, and three months after randomization. All data will be coded and entered into Excel files (Microsoft Corporation).

### Randomization

Patients who meet the eligibility criteria will be randomly allocated to their respective intervention groups.

### Allocation concealment

The individuals will be randomly allocated to the two groups. To minimize the risk of imbalance in the size of the groups, a randomization list will be generated using two blocks: number 1 for the manipulation group and number 2 for the placebo-controlled group. The allocation sequence will be stipulated in sequentially numbered, opaque, sealed envelopes. Following the baseline evaluation, each participant will be allocated to one of the groups by opening an envelope. This process will be performed by a member of the research team who is not involved in the recruitment process or other aspects of the study.

### Blinding

The design study of the trial does not allow blinding of the therapist. All the pre- and post-treatment assessments and the follow-up assessment will be done by a person blinded to group allocation and treatment. The statistician performing the statistical analyses will also be blinded to group allocation and treatment.

### Evaluation and follow-up

The evaluation process will be conducted by a physiotherapist with experience in the evaluation procedures and blinded to the allocation of the participants to the different groups. Evaluations will be conducted in the following manner:

- Pre-treatment evaluation
- Evaluation immediately following a single intervention session
- Post-treatment evaluation
- Evaluation three months after randomization

### Measurements

The scales to be administered are the NPRS, ODI, EMG signal (Biering-Sorensen test and Flexion-relaxation phenomenon), FFT, and Fear-Avoidance Beliefs Questionnaire (FABQ).

### Numeric Pain Rating Scale

The NPRS will be used to determine the level of pain intensity perceived by the patient using an 11-point scale, on which 0 represents the absence of pain and 10 represents the worst pain imaginable [35]. The participants will be instructed to report their sensation of pain intensity at the moment of the evaluation to compare with the immediate effect of treatment and to report average pain intensity based on the previous seven days for comparisons at the end of the six-week treatment and three-month follow-up.

### Oswestry Disability Index

The ODI is the most commonly used outcome measure for LBP. It is a self-administered questionnaire and each section is scored on a scale from 0 (no disability) to 5 (maximum disability). The index is calculated by dividing the sum of the item scores by the maximum possible score, which is then multiplied by 100 and expressed as a percentage. Thus, for every question not answered, the denominator is reduced by 5. If a patient marks more than one statement on an item, the higher scoring statement is recorded as a true indication of disability. The questionnaire takes 3.5–5 min to complete and approximately 1 min to score [36].

### Electromyographic analysis

#### Biering-Sorensen test

Before the FRP, all individuals will perform the Sorensen endurance test [37]. The prone position will be adopted with the trunk placed beyond the edge of the table, with the anterior superior iliac spine aligned with the edge of the table and the lower limbs fixed to the table. On this test, the patient maintains the horizontal position with the upper limbs crossed and in contact with the chest for 10 s, three times, with a 10-min rest after the third time [19, 21]. The maximum 1-s root mean square (RMS) activity recorded during the Sorenson test will be defined as the maximal voluntary contraction (MVC) value and will be used as a reference for other electromyographic data.

### Flexion-relaxation phenomenon

The EMG signal will be collected during this movement. The flexion/extension trunk movement will be started in the upright position. The participant will be instructed to move in response to voice command, keeping the knees straight but not locked, and the arms hanging freely, while slowly flexing forward to full flexion over a 3-s period, pausing for 3 s at full trunk flexion and then returning to the upright starting position during the 3 s of the trunk extension period. This protocol is typical of those used in studies on the FRP [17, 18, 21, 27].

The movement will be performed three times. Data from the third replication will be used in the analysis. Before the first reading, the patients will practice three times to become familiar with the movement [20, 21].

Two different forms of a flexion-relaxation ratio (FRR) will be used to quantify the degree to which the FRP is present [17, 18]. One will be calculated by dividing the maximum RMS of EMG activity level during flexion (while bending forward) by the lowest mean EMG activity as measured over a 1- interval during the fully flexed phase. Another FRR will be similarly calculated by dividing the maximum RMS EMG activity level during extension (while returning to the upright position) by the same minimum. The beginning and end of the fully flexed phase for each cycle will be determined from the plot of the motion data.

### Electromyographic signal

Electromyography is the most widely used assessment tool for the study of muscle activation during the FRP [17, 18]. A four-channel conditioning module (BTS FREEEMG 1000®) will be used with an A/D converter with 16-bit resolution, a common rejection mode ratio > 100 dB and 20–450 Hz bandpass filter. The EMG signals will be amplified with a 2000-fold gain using a 1-kHz sampling frequency and wireless transmission. The signals will be captured with self-adhesive, disposable, Ag/AgCl surface electrodes measuring 1 cm in diameter (Medi-Trace 200 Kendall Healthcare, Tyco, Canada). After cleaning the skin of the sites with 70% alcohol, the electrodes will be positioned at a distance of 2 cm center to center on the paraspinal muscles at T12 and L1 on each side with approximately 1 cm vertical distance between the edges of the electrodes in semi-flexed trunk position [21, 27]. The electrodes will not be removed during treatment, but the outline of each electrode will be made with a skin marking pen so that they can be placed in the same location for subsequent measurements if they become detached during treatment.

### Fingertip-to-floor test

The FFT will be performed during the third cycle of the FRP with full trunk flexion (static phase). The third finger of the dominant hand will be used [38]. The participants will stand on a platform measuring 30 cm in height to avoid touching the floor, which would make the measurement unviable.

### Fear-Avoidance Beliefs Questionnaire

The FABQ is a 16-item instrument used to determine a patient’s beliefs regarding the effects of physical activity and work on musculoskeletal pain. The responses for each item are scored on a seven-point scale (0 = completely disagree to 6 = completely agree). The original factor analysis revealed two subscales: a physical activity subscale with five items (maximum score = 24) and the work subscale with 11 items (maximum score = 42). The total is in the range of 0–96 points, with a higher score indicating more strongly held fear-avoidance beliefs. The FABQ takes about 10 min to complete [39].

### Statistical analysis

Statistical analysis will be performed using intention-to-treat analysis. If data losses occur during the study, the last observation will be carried forward to adjust the missing data in follow-up evaluations. The Shapiro–Wilk test will be used to determine the normality of the data. Anthropometric differences between groups will be determined using the independent t-test for data will normal distribution and the Mann–Whiney test for data with non-normal distribution. Repeated-measures analysis of variance (ANOVA) followed by the Bonferroni post hoc test will be used to determine the effects of treatment with regard to the NPRS, RMDQ, FFT, FABQ, and EMG considering the following interactions: group (OVM and SOVM) vs evaluation (pre-interventions, after one session, after six weeks, and three months after randomization) vs movement (flexion extension). If the data exhibit non-normal distribution, Friedman’s ANOVA will be used with Dunn’s post hoc test. A *p* value < 0.05 will be considered indicative of statistical significance. The data will be organized and tabulated using the Statistical Package for the Social Sciences (SPSS, v.19.0).

### Adverse events and safety

Adverse events (AEs) are recorded as part of the data collection for each session and will be reported to the clinical authorities and to the ethics committee. Participants suffering AEs will be referred for appropriate treatment.

### Compliance and blinding assessment

To assess patients’ blinding to treatment allocation, patients are asked post treatment (six weeks after the start of treatment) to report which study treatment they think that they received (OVM/SOVM). The effect of their reports on outcome will be examined in explorative analysis.

## Discussion

This paper presents a detailed description of a prospective, placebo-controlled, assessor-blinded, clinical RCT designed to demonstrate the effect of a six-week OVM program on the FRP in individuals with non-specific chronic LBP and functional constipation. It will also allow us to investigate neurophysiologic and biomechanical processes that may contribute to the therapeutic effects of OVM. Analyzing FRP measured in patients with LBP submitted to OVM may help clarify the contributions of passive and active structures during and following OVM, thereby providing evidence for suspected therapeutic mechanisms. The results will be published and the evidence found may contribute to the use of visceral manipulation for this population.

The results and practical relevance of our study will be of importance not only for researchers and policy makers but also for patients suffering from non-specific chronic LBP and functional intestinal constipation.

Given the nature of the study, the limitation of the study is that the therapist will not be blinded. Nevertheless, the design also has important strengths: reproducibility; and the blinding of the assessor and participant. The outcome will provide evidence-based conclusions regarding the effectiveness of this treatment for the management of patients with non-specific chronic LBP and functional constipation.

### Trial status

Participants will be recruited to start in January 2018. Data collection will be finished in May 2018 and study completion is expected to be July 2018.

## Additional file

Additional file 1: SPIRIT 2013 Checklist: Recommended items to address in a clinical trial protocol and related documents*. (DOC 122 kb)

## Abbreviations

ANOVA: Analysis of variance
CAPES: *Coordenação de Aperfeiçoamento de Pessoa de Nível Superior*
CONSORT: Consolidated Standards of Reporting Trials
EMG: Electromyographic
FAPEMAT: *Fundação de Amparo á Pesquisa do Estado de Mato Grosso*
FFT: Fingertip-to-floor test
FRP: Flexion-relaxation phenomenon
FRR: Flexion-relaxation ratio
LBP: Low back pain
MVC: Maximal voluntary contraction
NPRS: Numeric Pain Rating Scale
ODI: Oswestry Disability Index
OVM: Osteopathic visceral manipulation
RMS: Root mean square
SENIAM: Surface electromyography for non-invasive assessment of muscles
SOVM: Sham osteopathic visceral manipulation
SPSS: Statistical Package for the Social Sciences

## References

[1] Kamper SJ, Apeldoorn AT, Chiarotto A, Smeets RJ, Ostelo RW, Guzman J (2015). Multidisciplinary biopsychosocial rehabilitation for chronic low back pain: Cochrane systematic review and meta-analysis. BMJ.

[2] Nunes SK, Baptista AF, Matos MA, Lessa I (2008). Chronic pain and gender in Salvador population, Brazil. Pain.

[3] Hansson TH, Hansson EK (2000). The effects of common medical interventions on pain, back function, and work resumption in patients with chronic low back pain. Spine.

[4] Al-Eisa E, Egan D, Deluzio K, Wassersug R (2006). Effects of pelvic skeletal asymmetry on trunk movement: three-dimensional analysis in healthy individuals versus patients with mechanical low back pain. Spine (Phila Pa 1976).

[5] Koes B, Tulder M, Thomas S (2006). Diagnosis and treatment of low back pain. BMJ.

[6] Kent P, Keating JL (2005). Classification in nonspecific low back pain: what methods do primary care clinicians currently use?. Spine (Phila Pa 1976).

[7] Schneider MJ, Brach J, Irrgang JJ, Abbott KV, Wisniewski SR, Delitto A (2010). Mechanical vs manual manipulation for low back pain: an observational cohort study. J Manip Physiol Ther.

[8] Licciardone JC, Aryal S (2014). Clinical response and relapse in patients with chronic low back pain following osteopathic manual treatment: results from the OSTEOPATHIC Trial. Man Ther.

[9] Maigne J, Vautraver P (2003). Mode d’action dês manipulations vertébrales. Revue du Rheumatisme.

[10] Ernest E (2006). A systematic review of systematic review of spinal manipulation. J R Soc Med.

[11] Panagoupols J, Hancok M, Ferreira P, Hush J, Petocz P (2015). Does the addition of visceral manipulation alter outcomes for patients with low back pain? A randomized placebo controlled trial. Eur J Pain.

[12] McSweeney TP, Thomson OP, Johnston R (2012). The immediate effects of sigmoid colon manipulation on pressure pain thresholds in the lumbar spine. J Bodyw Mov Ther.

[13] Attali TV, Bouchoucha M, Benamouzig R (2013). Treatment of refractory irritable bowel syndrome with visceral osteopathy: short-term and long-term results of a randomized trial. J Dig Dis.

[14] Belvaux A, Bouchoucha M, Benamouzig R (2017). Osteopathic management of chronic constipation in women patients. Results of a pilot study. Clin Res Hepatol Gastroenterol.

[15] Tozzi P, Bongiorno D, Vitturini C (2012). Low back pain and kidney mobility: local osteopathic fascial manipulation decreases pain perception and improves renal mobility. J Bodyw Mov Ther.

[16] Tamer S, Öz M, Ülger Ö (2017). The effect of visceral osteopathic manual therapy applications on pain, quality of life and function in patients with chronic nonspecific low back pain. J Back Musculoskelet Rehabil.

[17] Neblett R, Mayer TG, Gatchel RJ, Keeley J, Proctor T, Anagnostis C (2003). Quantifying the lumbar flexion-relaxation phenomenon: theory, normative data, and clinical applications. Spine (Phila Pa 1976).

[18] Neblett R, Mayer TG, Brede E, Gatchel RJ (2010). Correcting abnormal flexion-relaxation in chronic lumbar pain: responsiveness to a new biofeedback training protocol. Clin J Pain.

[19] Ritvanen T, Zaproudina N, Nissen M, Leinonen V, Hänninen O (2007). Dynamic surface electromyographic responses in chronic low back pain treated by traditional bone setting and conventional physical therapy. J Manip Physiol Ther.

[20] Marshal P, Murphy B (2006). Changes in the flexion relaxation response following an exercise intervention. Spine.

[21] Bicalho E, Setti JA, Macagnan J, Cano JL, Manffra EF (2010). Immediate effects of a high-velocity spine manipulation in paraspinal muscles activity of nonspecific chronic low-back pain subjects. Man Ther.

[22] Finneran MT, Mazanec D, Marsolais ME, Marsolais EB, Pease WS (2003). Large-array surface electromyography in low back pain: a pilot study. Spine (Phila Pa 1976).

[23] Oddsson LI, De Luca CJ (2003). Activation imbalances in lumbar spine muscles in the presence of chronic low back pain. J Appl Physiol (1985).

[24] Colloca CJ, Hinrichs RN (2005). The biomechanical and clinical significance of the lumbar erector spinae flexion-relaxation phenomenon: a review of literature. J Manip Physiol Ther.

[25] Fryer G, Morris T, Gibbons P (2004). Paraspinal muscles and intervertebral dysfunction: part two. J Manip Physiol Ther.

[26] Demoulin C, Crielaard JM, Vanderthommen M (2007). Spinal muscle evaluation in healthy individuals and low-back-pain patients: a literature review. Joint Bone Spine.

[27] Xia T, Long CR, Vining RD, Gudavalli MR, DeVocht JW, Kawchuk GN (2017). Association of lumbar spine stiffness and flexion-relaxation phenomenon with patient-reported outcomes in adults with chronic low back pain – a single-arm clinical trial investigating the effects of thrust spinal manipulation. BMC Complement Altern Med.

[28] Airaksinen O, Brox JI, Cedraschi C, Hildebrandt J, Klaber-Moffett J, Kovacs F (2006). Chapter 4. European guidelines for the management of chronic nonspecific low back pain. Eur. Spine J.

[29] Miller LE, Ibarra A, Ouwehand AC, Zimmermann AK (2017). Normative values for stool frequency and form using Rome III diagnostic criteria for functional constipation in adults: systematic review with meta-analysis. Ann Gastroenterol.

[30] Manchester MR, Glasgow GW, York JKM (2002). The back book: clinical guidelines for the management of acute low back pain.

[31] Ricard F (2008). Tratado de osteopatía visceral y medicina interna osteopática: tomo 2: sistema digestivo.

[32] Verbeek J, Sengers M-J, Riemens L, Haafkens J (2004). Patient expectations of treatment for back pain: a systematic creview of qualitative and quantitative studies. Spine.

[33] Ostelo RW, De Vet HC (2005). Clinically important outcomes in low back pain. Best Pract Res Clin Rheumatol.

[34] Browder DA, Childs JD, Cleland JA, Fritz JM (2007). Effectiveness of an extension-oriented treatment approach in a subgroup of subjects with low back pain: a randomized clinical trial. Phys Ther.

[35] Costa LO, Maher CG, Latimer J, Ferreira PH, Pozzi GC, Ribeiro RN (2007). Psychometric characteristics of the Brazilian-Portuguese versions of the Functional Rating Index and the Roland-Morris Disability Questionnaire. Spine (Phila Pa 1976).

[36] Vigatto R, Alexandre NM, Correa Filho HR (2007). Development of a Brazilian Portuguese version of the Oswestry Disability Index: cross-cultural adaptation, reliability, and validity. Spine (PhilaPa 1976).

[37] Biering-Sørensen F (1984). Physical measurements as risk indicators for low-back trouble over a one-year period. Spine (Phila Pa 1976).

[38] Perret C, Poiraudeau S, Fermanian J, Colau MM, Ma B, Revel M (2001). Validity, reliability, and responsiveness of the fingertip-to-floor test. Arch Phys Med Rehabil.

[39] Abreu AM, Faria CD, Cardoso SM, Teixeira-Salmela LF (2008). The Brazilian version of the Fear Avoidance Beliefs Questionnaire. Cad Saude Publica.

